# Clustering of attitudes towards obesity: a mixed methods study of Australian parents and children

**DOI:** 10.1186/1479-5868-10-117

**Published:** 2013-10-12

**Authors:** Tim Olds, Samantha Thomas, Sophie Lewis, John Petkov

**Affiliations:** 1 Health and Use of Time (HUT) Group, University of South Australia, GPO Box 2471, 5001 Adelaide, SA, Australia; 2 School of Health and Society, Bldg 234 (iC Enterprise 1), Innovation Campus, University of Wollongong, Northfields Ave, 2522 Wollongong, NSW, Australia; 3 Faculty of Health Sciences, Cumberland Campus, University of Sydney, Sydney, Australia

**Keywords:** Obesity, Attitudes, Adults, Adolescents, Family, Social marketing

## Abstract

**Background:**

Current population-based anti-obesity campaigns often target individuals based on either weight or socio-demographic characteristics, and give a ‘mass’ message about personal responsibility. There is a recognition that attempts to influence attitudes and opinions may be more effective if they resonate with the beliefs that different groups have about the causes of, and solutions for, obesity. Limited research has explored how attitudinal factors may inform the development of both upstream and downstream social marketing initiatives.

**Methods:**

Computer-assisted face-to-face interviews were conducted with 159 parents and 184 of their children (aged 9–18 years old) in two Australian states. A mixed methods approach was used to assess attitudes towards obesity, and elucidate why different groups held various attitudes towards obesity. Participants were quantitatively assessed on eight dimensions relating to the severity and extent, causes and responsibility, possible remedies, and messaging strategies. Cluster analysis was used to determine attitudinal clusters. Participants were also able to qualify each answer. Qualitative responses were analysed both within and across attitudinal clusters using a constant comparative method.

**Results:**

Three clusters were identified. *Concerned Internalisers* (27% of the sample) judged that obesity was a serious health problem, that Australia had among the highest levels of obesity in the world and that prevalence was rapidly increasing. They situated the causes and remedies for the obesity crisis in individual choices. *Concerned Externalisers* (38% of the sample) held similar views about the severity and extent of the obesity crisis. However, they saw responsibility and remedies as a societal rather than an individual issue. The final cluster, the *Moderates*, which contained significantly more children and males, believed that obesity was not such an important public health issue, and judged the extent of obesity to be less extreme than the other clusters.

**Conclusion:**

Attitudinal clusters provide new information and insights which may be useful in tailoring anti-obesity social marketing initiatives.

## Background

Obesity is considered one of Australia’s most significant public health issues with a marked increase in the prevalence of obesity over the past 30 years [1]. About 25% of adults are obese with a further 37% overweight [1]. About 20-25% of Australian children and adolescents are overweight or obese [2]. Obesity has a range of negative health, social and financial outcomes for individuals, families, communities and society. It is a risk factor for Type 2 diabetes, cardiovascular disease, some cancers, and some musculoskeletal and respiratory conditions [3]. In 2008, it was estimated that the overall financial cost of obesity to Australian society and governments was $58 billion [4]. The stigma associated with being obese may also have a serious negative impact on individuals’ health and wellbeing. Feelings and experiences of weight bias and stigma may contribute to depression, anxiety, low self-esteem, reduced social support and social isolation [5,6].

More recently, researchers have shown that prevalence of overweight and obesity among children have plateaued in Australia [2,7]. While these are encouraging findings, public health and health promotion practitioners remain committed to finding more effective ways to prevent and manage overweight and obesity. The causes of obesity are undoubtedly complex and require a range of different ‘solutions’ [8], and social marketing initiatives are an important part of the arsenal of strategies used by government and community-based organisations to encourage behavioral change [9].

To date, most of these initiatives have been focused on ‘downstream’ patterns of communication (most regularly through media based campaigns) that try to persuade individuals to take ‘personal responsibility’ for their (over)weight. Most of these campaigns are targeted to individuals according to their weight characteristics (e.g. overweight or obese) or socio-demographic characteristics (e.g. parents, children, men, or different age groups). For example, the Australian based ‘Measure Up’ campaign encourages “people to make positive lifestyle changes (specifically in the areas of nutrition and physical activity)” and as a result “reduce the prevalence and impact of chronic disease on the Australian community”, with a key target audience of parents aged 25–50 years [10].

However, there is debate about how effective these types of campaigns are in changing individual behavior and attitudes towards obesity. Some researchers suggest that if social marketing campaigns are to effectively respond to, and prevent, major social issues such as obesity, they must look to target a broader audience than just “problem people” [11] p. vii, and should shift from mass messages about personal responsibility, towards more sophisticated attempts to influence behavioural change [12-14]. This includes using public opinion to influence policy and practice.

Some studies suggest that this lack of sophistication in social marketing campaigns, including an overwhelming focus on individual responsibility framing, may partly explain why campaigns have had only minimal positive effects on both short and long term attitudes and behavior, and may have unforeseen or unintended consequences [15]. As such, researchers now argue that it is important to consider how a more sophisticated set of social marketing strategies may be developed for obesity that, a) take into account socio-demographic and weight characteristics, but b) resonate with how different types of people conceptualise weight and health. One way of approaching this is to explore the beliefs held by different groups of individuals about the causes of and solutions for obesity, and to design campaigns that respond to these beliefs and behaviours. These types of studies are not only important in tailoring ‘downstream’ social marketing campaigns (which target individuals), but may also be useful in developing ‘upstream’ strategies which seek to shift obesity policy. For example, it may be possible for social marketers to use knowledge about existing clusters of attitudes and behaviours, in particular audience segments, to advocate for changes in obesity policy, food industry practices, or the media reporting of obesity. Alternatively, social marketing may be used to target clusters of individuals who may be misinformed about the causes and consequences of obesity as a first step to then help individuals change behaviours, or to reduce the stigmatization of obese adults.

To date, only a small number of studies have explored how public opinions about obesity are formed, including public attitudes about the causes, consequences and solutions for obesity [16-18], and how these opinions may be used to inform social marketing initiatives [19]. These studies show that public concern about obesity has increased, with a perception that obesity is an escalating problem for communities [17,20]. However, increasing public concern about the health and social consequences of obesity has not necessarily translated into changing attitudes towards obesity policy and interventions. Obesity is still largely regarded as an issue of personal (or parental) (ir)responsibility [17,20-23], with some researchers stating that this has led to lower support for broad-based prevention initiatives [24]. Beliefs about the causes of obesity may influence people’s beliefs about the appropriate solutions for obesity, including support – or lack of – obesity policy and regulation. For example, Barry and colleagues [16] found that individuals – independent of weight status – who endorsed societal or environmental causes of obesity such as “a toxic food environment” and “industry manipulation”, were more supportive of policy action, while those who believed that obesity was caused by individual choices were less supportive of reform [16]. Similarly, Oliver and Lee [18] found that beliefs about the causes and consequences of obesity were stronger predictors of support for obesity policy than socio-demographic factors or an individual’s weight or diet and exercise profiles. Finally, studies show a link between individuals’ beliefs about the causes of obesity and their health behaviours. Individuals who believe obesity is caused by genetic predisposition report lower levels of physical activity and fruit and vegetable consumption, than those who believe that obesity is the outcome of inactivity and overeating [25].

Many researchers now argue that further empirical research is needed to understand how these attitudes are clustered in particular population subgroups, what leads to these attitudes, and how attitudes may be changed [24].

This study aims to identify the clustering of attitudes about obesity in Australian families, and to describe how we can use this information to move away from ‘one size fits all’ approaches to social marketing efforts aimed at combating obesity, towards strategies that are tailored to resonate with the attitudes and opinions of different population subgroups.

## Methods

### Approach

We employed a mixed methods approach for this study, and utilised a concurrent embedding approach to understand attitudes toward obesity [26]. This approach had quantitative techniques as the primary method within an interviewer-assisted survey, with qualitative questions providing a “supporting role” within the survey to understand why individuals held various beliefs and opinions towards obesity and any influences that may have informed these opinions. As described by Cresswell [27] the aim was to bring together two different pictures about attitudes towards obesity, which could then be used to develop an overall assessment of what attitudes exist and how they are formed. In this study, we chose to focus on families because we were interested in attitudinal differences and similarities between parents and their children. This is a unique addition to current literature on attitudinal clusters which focuses predominantly on the clustering of adult attitudes towards obesity and obesity policy.

The study was approved by the Human Research Ethics Committees of the University of South Australia and Monash University.

### Recruitment

Families (at least one adult and one child between the ages of 9–18) were recruited via two professional recruiting companies in Victoria and South Australia, with funding from the Australian Research Council Discovery Grant Scheme. To ensure that the sample covered a wide range of socio-economic positions, recruitment occurred in equal numbers across socio-economic tertiles defined by the Socio-Economic Indicators for Areas Index of Relative Social Disadvantage (IRSD). The IRSD is a postcode-level measure of socio-economic position based on a basket of indicators such as household income and parental education. We chose to sample by IRSD because of Australian literature that suggested that there were key differences in weight and health outcomes based on socio-economic status [28]. Non-English speakers, children under nine years of age, and those living in remote geographical locations were excluded.

### Data collection

Each family took part in an audio-taped face-to-face interview (lasting between 45 and 120 minutes) with two trained researchers. One researcher conducted the interview, while the other took notes about family dynamics, responses, and anything else of interest from the interview. At the completion of the interview each family was reimbursed with a $100 grocery or petrol voucher for their participation.

For the survey, parents and children were separated and interviewed in different spaces of the house by two different interviewers to ensure that parents did not influence children’s responses (and vice versa). At no stage were parents present during the child’s interview, although children in the same family were interviewed together, and we cannot be sure that siblings did not influence each others responses. All parents and children completed the survey. Parents and children were told by the interviewers that there were no right or wrong answers, and that we were only interested in their opinions. All interviews were audio-taped. Two interviewers were present and one interviewer conducted the interview while the other interviewer recorded notes about anything associated with interaction within the family. Both interviewers had ‘debriefing’ sessions where these notes were discussed on the day of the interview, and any other ideas or thoughts about the interview were recorded. These were then discussed at the broader team meetings amongst the study investigators. Notes were used to understand the more subtle interactions between the family and were used to supplement the material collected in the interview. Participants first completed a brief socio-demographic survey, reporting their gender, age, height and weight (from which body mass index and weight status were derived using International Obesity Task Force criteria [29]; and for adults, educational status (coded as not finished high school, completed high school, post-secondary diploma, or university); ethnicity (coded as Australian, European or other); and marital status (married or de facto, single, separated, divorced or widowed); income; occupation; and family structure. Participants then rated their attitudes on seven questions using a 0–10 scale (Table 1), and were asked to estimate the percentage of Australian adults who were obese. Questions were designed to elicit responses in relation to the major attitudinal dimensions around obesity: the severity of the issue; prevalence and trends; individual vs. societal responsibility; genetic vs. lifestyle causal factors; and strategies to address obesity. These dimensions were derived from a review of the literature, the popular media [30] and earlier qualitative studies by this group [31,32].

**Table 1:** Questions used to quantify attitudes towards obesity and weight management

**Title**	**Question**	**Verbal anchor for 0**	**Verbal anchor for 5**	**Verbal anchor for 10**
**Severity**	Do you think obesity is a serious and major health problem in Australia?	Not a problem at all, just hype	A problem, but not as serious as some say	It’s Australia’s most serious health problem
**Rank**	Where do you think Australia ranks in the global prevalence of obesity?	In the bottom half	In the top 10 fattest nations	Australia is the fattest nation
**Trends**	Do you think more and more people are becoming overweight and obese?	No, there’s been no increase in the last decade	It seems to be still increasing	It’s increasing faster than ever
**Cause**	What do you think is the main cause of obesity?	Almost completely by genetics	A combination of genetic and lifestyle and environmental factors	Lifestyle and environment
**Blame**	Who do you think is to blame for childhood obesity?	Entirely the fault of parents and children	An equal mix of both	It’s all because of the society we live in
**Remedy**	What do you think is the best way to reduce obesity?	People have to change themselves	Make the right choice the easy choice	Force people to change. Make it impossible to eat bad food and be inactive
**Messages**	What is the best way to get people to lose weight: fear and shame, or positive messages?	Fear and shame work for tobacco: they should work for obesity too	Maybe a bit of both	We should focus on healthy eating and physical activity
**Prevalence**	What percentage of Australian adults would be considered “obese”?	0	50	100

Finally, participants were asked open-ended questions about how often (if at all) they discussed issues related to weight, who were involved in these discussions, and what was discussed. They were also asked whether they were currently trying to gain or lose weight, whether they had been successful, and what methods they used to change their weight.

### Analysis

Medians, means and standard deviations were calculated for responses to each of the eight questions. Spearman’s rho was used to quantify the strength of the associations among the responses. Children’s and parents’ responses were compared across the eight attitudinal dimensions using Mann–Whitney tests. Participants were clustered according to the eight attitudinal variables (Table 2). All variables were standardised before clustering. Analysis was performed using K-Means clustering based on squared Euclidean distance [33]. The number of clusters was determined from an error function plot. Centroids were calculated and cluster membership was determined based on the nearest centroid. Feature Selection, a data mining approach [34], was used to determine which input variables were the most important in discriminating among the clusters. Cluster correlates were determined using analysis of variance for continuous variables, and chi-square for categorical variables. Alpha was set at 0.05, and sequential Bonferroni adjustment was used to correct for capitalisation on chance.

**Table 2:** Characteristics of the participants in this study

		**Parents**	**Children**^**a**^
**N**		159	184
**% Female**		82	50
**Age (years)**		44.7 (6.0)	13.5 (2.7)
**BMI (kg.m**^**–2**^**)**		28.4 (6.7)	20.0 (3.5)
**Weight status**	**% overweight**	35	18
	**% obese**	31	4
**Education**	**University**	23	
	**Diploma**	30	
	**Year 12**	24	
	**Year 10**	22	
**Ethnic background**	**Australian**	68	
	**European**	28	
	**Other**	4	
**Marital Status**	**Married/de facto**	82	
	**Separated/divorced/widowed**	12	
	**Single**	6	

^a^The word “children” will be used to refer to both children and adolescents.

BMI = Body mass index.

We then analysed the qualitative questions that were embedded within the survey using a constant comparative method [35], and compared them to the quantitative results. The aim of this was to provide a rich narrative complement to the statistical data and to help us to understand why and how attitudes had been formed. Participants’ narratives were grouped according to the three attitudinal clusters. We then compared participants’ qualitative responses to their quantitative responses, looking for differences and similarities in responses, and differences and similarities in responses within and between participant groupings.

## Results

### General characteristics of the sample

A total of 159 parents and 184 children and adolescents (aged 9–18 hereinafter referred to as children) in two Australian states (75 families in Victoria and 75 families in South Australia) participated in this study. Table 2 shows the characteristics of the participants. Their self-reported weight status, ethnic background, education and household composition were broadly similar to the general Australian population [2,36,37] (Table 2).

### Attitudinal variables

Table 3 shows the descriptive data for the attitudinal variables. The participants in this study considered obesity to be a major public health issue (with a median rating for Severity of 7), felt that Australia was among the ten fattest nations or higher [median rating for Rank = 7; Australia is currently ranked about #21 [38], and that obesity was increasing rapidly [median rating for Trends = 6; the prevalence of obesity appears to be increasing in Australia, but at a somewhat slower rate than in the past [2]. Participants estimated that 44 ± 20% of Australian adults were obese (the current figure is 20-25%). While these judgments demonstrate a degree of catastrophisation of the obesity issue, there was a wide range of views. While some rated Severity as 10, others rated it as 3. Estimates of Prevalence ranged from an optimistic 0% to a catastrophic 95%.

**Table 3:** **Medians, means and standard deviations for the eight attitudinal dimensions outlined in Table **1**, for parents (n = 159), children (n = 184) and all participants (n = 343)**

	**Parents (n = 159)**				**Children (n = 184)**				**All participants (n = 343)**				
**Dimension**	**Median**	**Mean**	**SD**	**Range**	**Median**	**Mean**	**SD**	**Range**	**Median**	**Mean**	**SD**	**Range**	**P**
**Severity**	7	7.7	1.3	4-10	8	6.7	1.6	3-10	7	7.2	1.5	3-10	<0.001
**Rank**	7	6.5	1.8	1-10	5	5.7	2.5	0-10	7	6.1	2.3	0-10	<0.001
**Trends**	7	6.8	1.8	0-10	6	6.0	2.0	0-10	6	6.3	2.0	0-10	<0.001
**Cause**	7	7.2	1.6	2-10	7	6.8	2.0	0-10	7	7.0	1.8	0-10	0.02
**Blame**	5	4.4	2.0	0-10	5	5.21	1.9	0-10	5	4.9	2.0	0-10	<0.001
**Remedy**	5	3.9	2.1	0-10	5	4.1	2.1	0-9	5	4.0	2.1	0-10	0.29
**Messages**	8	8.0	1.9	2-10	7	6.9	2.2	0-10	8	7.4	2.1	0-10	<0.001
**Prevalence**	45	44	19	1-90	44	43	21	0-95	45	44	20	0-95	0.58

Each dimension was scored on a 0–10 scale. The P-values refer to a comparison of parent and child means.

There were a wide range of opinions relating to the causes of and remedies for obesity. In relation to Cause, participants felt lifestyle and environment, rather than genetics, were the root cause of obesity (median rating 7). Participants were undecided whether individuals (as opposed to the broader society, including environment and technology) were responsible for their own obesity (median rating for Blame = 5). They were also ambivalent as to whether remedies rested with the individual or the society as a whole (median rating for Remedy = 5). There was however very strong opposition to the idea of forcing people to change through tobacco-style fear and shame campaigns, in favour of positive messages targeting individual choice (median rating for Messages = 8). Again, there was a very wide spread of views on all these questions, with ratings ranging from 0 to 10.

Children scored significantly higher on Severity, Trends and Blame, but lower on Rank, but lower on Rank, Cause and Messages.

### Correlations among the attitudinal dimensions

Correlations among the ratings on each of the eight attitudinal dimensions were calculated across all participants. There were weak to moderate, but significant, positive correlations among estimates of the Severity, Rank, Trends and Prevalence of obesity (rho = 0.18-0.38). There were also weak and significant positive correlations between estimates of Severity, Rank and Trends on the one hand and Cause on the other (rho = 0.17-0.23). Participants who felt that obesity was a more severe and pressing problem also felt that lifestyle and environment, rather than genes, were responsible for the high prevalence of obesity. There were no significant correlations among the other attitudinal variables.

### Cluster analysis

Cluster analysis identified three clusters (Table 4). We labeled participants in the first cluster *Concerned Internalisers (CI)* (n = 93). These participants judged that obesity was a serious health problem, that Australia was among the fattest nations and that prevalence was rapidly increasing. Qualitative responses indicated that CI beliefs about the severity of the obesity epidemic in Australia were based on: 1) media reporting of the obesity epidemic in Australia; 2) comparing Australia to countries that they believed were ‘fatter’ e.g. the United States; and 3) personal observations based on what they had ‘seen’ in their communities – particularly in relation to childhood obesity. For example one parent stated that she believed the rate of obesity in Australia was becoming more serious because she had read a media report that more children were visiting their doctors for problems with weight at a younger age:

Just today the paper was talking about children, ten year olds weighing far more than they should and four year olds coming into to see doctors. (Female, 45 years old, Married/de facto)

**Table 4:** Centroids for the three clusters

**Attitudinal variable**	**Cluster 1:**	**Cluster 2:**	**Cluster 3:**
	**“Concerned**	**“Concerned**	**“Moderates”**
	**Internalisers”**	**Externalisers”**	
**N (%)**	93 (27%)	130 (38%)	120 (35%)
**Severity**	7.8	7.9	5.9
**Rank**	7.1	7.0	4.3
**Trends**	7.0	7.1	5.0
**Cause**	7.6	7.1	6.3
**Blame**	3.1	5.7	5.2
**Remedy**	2.2	5.3	3.9
**Messages**	7.4	7.6	7.2
**Prevalence**	45	55	31

She went on to state that because the amount of information about obesity had increased considerably, she assumed that the rates of obesity must also be increasing, particularly in children:

It must be increasing faster than ever because the fact that there is a lot more information out there. There are a lot more kids and people suffering Type 2 Diabetes. (Female, 45 years old, Married/de facto)

Quantitative analysis revealed that CIs also placed the blame for the obesity crisis on individuals — parents and children — and felt that the solution to the obesity crisis lay with individuals. Qualitative responses from CI children revealed that they were at times defensive of the food industry “I don’t reckon they have to blame business”, and thought that it was the fault of the individual if they consumed too much unhealthy food, and that individuals should exercise more self-control:

It’s so stupid. It’s your choice if you want to buy it and if you get overweight or obese it’s your fault. It’s [the fast food industry] business but it’s your choice whether you go in. They may be advertising but it’s temptation. If you go in you go in. You’re just giving them more business. (Female, 17 years old)

Parental responsibility, and most commonly control over fast food choices, was also a very strong theme within the qualitative responses of CI parents and children. A few CI children stated that parents should have more control over children’s food choices and demands:

I think it’s the parents’ fault. If the kids see an ad on TV and ask the parents ‘can I go and get a McDonalds?’, the parents don’t have to say yes. (Female, 11 years old)

Some of the CI parents held themselves up as an example of someone who had invested considerable time and energy into educating their children about making the ‘right’ food choices. While this participant was critical of the advertising strategies of the food industry (s)he still believed that parents should make the correct choices for their families:

The parents buy the food and the parents are educating the child about what’s healthy. With my children, it is a slow process to teach them to eat healthily. You’ve got to be creative. It is about the parenting. Certainly the food companies, the advertising in the morning during the children’s programs is terrible. But parents could turn the TV off. It’s all about the parents in those early years. It’s the parents [who are] to blame. Parenting is that, it’s a doing word. It’s entirely the parents’ responsibility. (Female, 52 years old, Married/de facto)

We labeled the members of the second cluster ‘*Concerned Externalisers’ (CE)* (n = 130). This group also judged obesity to be a serious issue, and believed that the prevalence of obesity in Australia to be very high and increasing. Their estimate of the percentage of people who were obese in Australia (55%) was the highest of all the clusters. Qualitative responses showed that many adults within this cluster perceived obesity to be a serious prospect for Australia. Adults and children both described the rapid increase in obesity. These perceptions were influenced by more reports about obesity in popular media, and a perceived increase in the advertising of unhealthy foods. However, as with ICs many individuals used the United States as a barometer for Australia’s ‘fatness’. Children sometimes felt that Australia was a ‘fat nation’ because we were a wealthy nation, and related obesity to overconsumption:

Well America’s the fattest because that’s where the capital city of junk food is. We’re probably pretty high up considering we are a rich country. We’re not in the bottom because that’s where Africa and places like that are. (Female, 13 years old)

Unlike the CIs, however, this cluster placed the blame for obesity on society as a whole, and felt that addressing obesity was as much a societal as an individual issue. Qualitative data showed that they were more focused on the broader societal costs associated with obesity, for example the increasing burden on the health care system. Adult CEs also thought about the long term consequences of obesity, and were prevention-focused. For example, some adults stated that governments had a “moral duty” to act or intervene:

I think that in the long run, later down the track they’re [the government] going to be paying out for Medicare and people to have weight loss surgery and for medical bills anyway. So I think the more they act on it, the less they’ll be paying in the long run. (Female, 27 years old, Married/de facto)

Although child CEs described regulation and the need for government to take “leadership” in tackling obesity, they also emphasised personal responsibility:

If people know that they look like Oompa Loompas then they maybe shouldn’t eat the food. And like helping them as well, showing them what’s going to happen in ten years’ time. Some people might still want to eat a little bit of junk food but we still need to sort of regulate a little bit. (Female, 13 years old)

CEs’ attitudes about government intervention and personal responsibility were strongly shaped by their own personal experiences. For example, some felt divided about the causes of obesity, stating that while fast food marketing tactics encouraged the consumption of unhealthy products, people needed to be educated to make more appropriate choices. However, some in this group also used personal examples to show how difficult it was for people to resist the consumption of these products “we are a fast food society… if I don’t have time to cook it is quick and easy”, while others stated that governments needed to more strongly use regulation to encourage personal responsibility:

I think some of the reasons that there are problems is because … [the Government] [hasn’t] regulated enough in the first place. They’ve just looked too much at the profit or the business side of companies making money by producing food in ways that …produce unhealthy food. So I think they’re equally responsible for perhaps the problems and the lifestyle that people lead nowadays. (Female, 53 years old, Separated/Divorced/Widowed)

We labeled the final cluster the ‘*Moderates*’ (M) (n = 120). *Moderates* felt that obesity was not such an important public health issue, and judged the prevalence of obesity (31%), Australia’s global ranking and the rate of increase to be less extreme than CIs and CEs. In relation to individual and societal responsibility, they held intermediate views to the other clusters. However, the qualitative data showed that were slightly different reasons for these moderate views between children and adults. Some adult Ms were more questioning and skeptical about the information they received about obesity, particularly from the media and their health professionals. Another mother stated that a doctor’s assessment of her weight was out of line with how she viewed her body:

I was told that I’m obese which I find that bizarre. Yes alright I’m carrying a bit of weight, like a *little bit* of weight, but I’m definitely not obese. But on the doctor’s scales because of my height I’m obese. I’d like to lose weight for my own self but I’m not obese. It does make you feel like crap to be told that you’re obese. And my husband’s obese, so we say ‘okay we’ll be obese together’. (Female, 37 years old, Married/de facto)

However, the views of a few child Ms appeared to have been subtly influenced by industry. For example, the following child stated:

I wouldn’t say that [we are one of the fattest] because we went to the Cadbury [chocolate] factory in Tasmania and I don’t think we’re in the top ten. (Male, 12 years old)

Another stated that he perceived that obesity was predominantly genetic or the result of parental role modeling because he had noticed that:

**…**everyone that I’ve seen at Macca’s and like that, if the kids are fat the parents are fat. It might not be genetics but the parents show what they’re doing and the kids want to be like that as well so they’ll just eat along with them. (Male, 12 years old)

**Figure 1:**
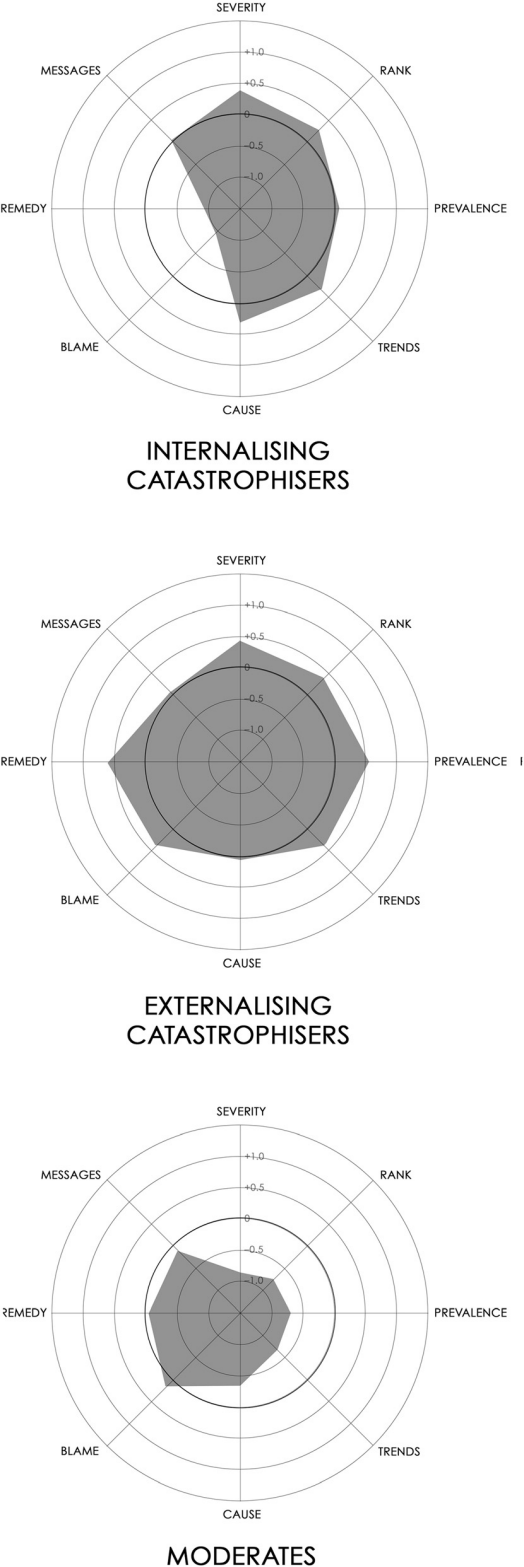
**Radar graphs for the three clusters.** The eight attitudinal dimensions are represented by the spokes of each graph. The concentric circles −1.0, –0.5, 0, +0.5 and +1.0 standard deviations away from the grand mean for each dimension.

Figure 1 shows radar graphs for the clusters. The most dissimilar clusters were the *Concerned Internalisers* and the *Moderates* (normalised Euclidean distance = 0.30 units), and between the *Concerned Externalisers* and the *Moderates* (0.29 units). The distance between the two Concerned clusters was smaller (0.18 units).

### Cluster discriminants

Feature Selection showed that all input variables except Messages were significant (p < 0.001) discriminants among the clusters. The most powerful predictors in order were: Remedy, Severity, Rank, Blame, Trends, Prevalence and Cause.

### Cluster correlates

Cluster membership was not associated with weight status, ethnicity, education, marital status, or the state where the interviews took place (Victoria vs. South Australia). There were, however, significant associations with age and sex. Cluster 3, the *Moderates*, had a significantly (p < 0.001) lower percentage of adults (26%) than the other clusters (63% and 53%). The *Moderates* cluster also had a significantly (p = 0.02) higher percentage of males (42% vs. 34% and 27%). Finally, cluster membership was associated with whether participants had ever discussed weight management issues (p = 0.02), with fewer *Moderates* (58%) than *Concerned Internalisers* (73%) or *Concerned Externalisers* (76%) reporting discussing weight management.

## Discussion

Before discussing the findings of this study it is important to recognize some limitations. Firstly, it is possible that due to perceptions of stigma, parents with very obese children may have been discouraged from participating in the study. However, based on self-report, the percentage of children (22%) and parents (65%) who were overweight or obese was similar to national data [2,36]. The proportions of respondents who were married (83%), separated, divorced or widowed (11%) and single (6%) were also similar to national data for families. Secondly, the majority of the participants in this study were white Australian, and may not reflect the views of a culturally and linguistically diverse group of families. In addition, the study was conducted with families who live in metropolitan areas, and future research will provide important insights into how rural families view and respond to obesity. Finally, one of the attitudinal dimensions (Cause) failed to distinguish between “lifestyle” and “environment”, since the concept being explored was individual as opposed to communal attribution. In retrospect, it would have been interesting to tease out these two aspects.

The main finding of this study was that the views of Australian parents and their children towards obesity fell into three roughly equal attitudinal clusters, based on their assessment of the severity of the obesity issue, and on the causes and remedies. Both *Concerned Internalisers* and *Externalisers* felt that obesity was a very serious problem, but differed as to whether responsibility was primarily personal or societal. *Moderates* felt that obesity was not such a serious issue. Interestingly, these clusters had very few socio-demographic correlates. *Moderates* tended to be younger and were more likely to be males, but weight status, educational level, ethnicity, marital status and geographical status were not associated with cluster membership. This suggests that traditional methods of socio-demographic market segmentation may not be applicable in the obesity domain.

This paper raises three key points for discussion. Firstly this study shows that there are a diverse range of views about and attitudes towards obesity. While there appeared to be a clear uptake of the ‘key messages’ about obesity (including healthy eating and activity), there also appeared to be significant polarization of views about the obesity problem. For example, some catastrophised the problem by confusing overweight and obesity, and holding unrealistic views about the extent and the rapidity of the increase in rates of obesity. Others were relatively unconcerned about the long term health and social impacts of obesity. This finding is perhaps unsurprising given the competing and at times confusing range of messages that are given about obesity from a range of different agencies. Previous studies have highlighted the inaccurate and at times exaggerated information that is communicated about obesity from the media, academics, industry and government [39,40], while research into public health campaigns suggests that individuals may be ‘shutting off’ from obesity messaging strategies which are increasingly based on ‘fear’ and which they consider at best to be irrelevant for their needs and at worst stigmatizing [24,41]. Without a clear understanding of how and why attitudes cluster within different groups, and how and why attitudes may change over time, it will be difficult for public health practitioners to create salient social marketing strategies – that is, messages that are relevant and significant to the target audience [42,43]. An important part of any social marketing initiative is to understand the range of cultural and environmental factors that may influence the way in which an individual, or groups, receive, interpret and apply messages about, in this case, obesity. Further research will provide important information about how individuals form their opinions, and any factors that may lead to disconnectedness between groups of individuals and messages that are given about weight and health [44].

An important area for further investigation is to examine how and why attitudes towards obesity may change from child to adulthood, and how any attitudinal changes may affect health behaviours. Children in this study were less likely to catastrophise the causes and consequences of obesity than adults, and favoured societal rather than individual responses to obesity. These attitudinal differences may be partly caused by the different ways in which information about obesity is communicated to different groups. At present, public health and health promotion strategies which aim to tackle childhood obesity are based on more collective, community and social approaches to weight and health outcomes. For example, the Australian Government has introduced a number of community-based childhood obesity campaigns including the ‘healthy communities initiative’ (introduced in 2009–10) which provides grants to local government areas to support local, community-based healthy eating and physical activity programs; and the Stephanie Alexander Kitchen Garden National program (introduced 2008) to promote healthy food choices among children. In contrast the vast majority of social marketing campaigns for adults are based on weight status and personal responsibility frameworks for change [45]. Similar to other studies [24] we found that most adults were supportive of public responses to prevent obesity. While not a community-wide phenomenon, a substantial minority of the sample (27%) believed that individuals were personally responsible for causing and resolving this issue. As such we would argue that there should be a more concerted effort by public health practitioners to create a more balanced spectrum of messages which cover both individual and collective measures (such as advocating against the marketing of unhealthy foods and beverages towards children, or engaging in developing community programs to encourage physical activity). This, in our opinion, could help to create a cultural shift in public attitudes among a section of the population, away from an overemphasis on personal responsibility (which may inherently remove the responsibility from industry), and towards a more collective response to healthy lifestyles. It may also help to activate social network-based initiatives – such as natural helper networks, which are able to both deliver and actively advocate for social change [46].

While there have been other studies which have examined public attitudes and opinions towards obesity and obesity policies [47] our study adds important additional data by exploring how these attitudes and opinions are constructed within a family setting, and how people cluster around a diverse range of attitudes and opinions about obesity. This study has highlighted the importance of understanding how different beliefs and attitudes may cluster into groups in the community, and how this in turn may impact on obesity related behaviours. These types of studies allow us to move away from a ‘one size fits all’ approach to obesity, to a more sophisticated understanding of how to approach clusters of individuals with different obesity related health beliefs and behaviours. Randolph and Viswanath [48] have highlighted the importance of culture in influencing individuals: *“health-related priorities, decisions, behaviors, and/or with acceptance and adoption of health education and health communication programs and messages”*. Although many public health policy initiatives are trying to tackle the broader socio-determinants of health, the majority of obesity messaging strategies targeted at adults are based on individualistic strategies for change. Many academics have made compelling arguments for the need to consider issues of personal responsibility within broader socio-ecological frameworks of health [49]. Communities are both the context for, and drivers of, behavioural change, and messages that engage with communities rather than individuals will be instrumental in encouraging and supporting the uptake of social marketing initiatives [50].

## Conclusion

Attitudinal clusters are important in the development of more effective social marketing strategies that seek engage communities in tackling the social rather than individual determinants of obesity. Without a clear understanding of how and why attitudes cluster within different groups, and how and why attitudes may change over time, it will be difficult for public health practitioners to create salient social marketing strategies – for example, messages that are relevant and significant to the target audience. Further research which examines how attitudes develop and are reinforced, could help social marketers create anti-obesity strategies that have greater saliency with diverse audiences and social contexts; encourage a balance of individualistic and collective approaches to health; effectively counterframe a diverse and often influential range of industry messages; and gain community support for a diverse range of regulatory and policy responses.

### Consent

Written informed consent was obtained from the guardian/parent/next of kin of each participant under the age of 18, and from each participant aged 18 or over for the publication of this report. Verbal assent was also obtained from each participating child.

## Competing interests

The authors declare that they have no competing interests.

## Authors’ contributions

TO & ST conceived the study. TO contributed to the study design, and advised on data analysis. ST led the analysis and the writing of the manuscript. SL contributed to the study design. JP completed the statistical analysis. All authors contributed to the interpretation of the findings and critical revision of the manuscript. All authors read and approved the final manuscript.
